# Summary of methodology used in enterotoxigenic *Escherichia coli* (ETEC) challenge experiments in weanling pigs and quantitative assessment of observed variability

**DOI:** 10.1093/tas/txad083

**Published:** 2023-07-20

**Authors:** Payton L Dahmer, Joel M DeRouchey, Jordan T Gebhardt, Chad B Paulk, Cassandra K Jones

**Affiliations:** Department of Animal Sciences and Industry, Kansas State University, Manhattan, KS, USA; Department of Animal Sciences and Industry, Kansas State University, Manhattan, KS, USA; Department of Diagnostic Medicine/Pathobiology, Kansas State University College of Veterinary Medicine, Manhattan, KS, USA; Department of Grain Science and Industry, Kansas State University, Manhattan, KS, USA; Department of Animal Sciences and Industry, Kansas State University, Manhattan, KS, USA

**Keywords:** Enterotoxigenic E. coli, sample size, variability, weanling pig

## Abstract

Postweaning diarrhea in pigs is often caused by the F4 or F18 strains of enterotoxigenic *Escherichia coli* (**ETEC**). To evaluate interventions for ETEC, experimental infection via a challenge model is critical. Others have reviewed ETEC challenge studies, but there is a lack of explanation for the variability in responses observed. Our objective was to quantitatively summarize the responses and variability among ETEC challenge studies and develop a tool for sample size calculation. The most widely evaluated response criteria across ETEC challenge studies consist of growth performance, fecal consistency, immunoglobulins, pro-inflammatory cytokines, and small intestinal morphology. However, there is variation in the responses seen following ETEC infection as well as the variability within each response criteria. Contributing factors include the type of ETEC studied, dose and timing of inoculation, and the number of replications. Generally, a reduction in average daily gain and average daily feed intake are seen following ETEC challenge as well as a rapid increase in diarrhea. The magnitude of response in growth performance varies, and methodologies used to characterize fecal consistency are not standardized. Likewise, fecal bacterial shedding is a common indicator of ETEC infection, but the responses seen across the literature are not consistent due to differences in bacterial enumeration procedures. Emphasis should also be placed on the piglet’s immune response to ETEC, which is commonly assessed by quantifying levels of immunoglobulins and pro-inflammatory cytokines. Again, there is variability in these responses across published work due to differences in the timing of sample collection, dose of ETEC pigs are challenged with, and laboratory practices. Small intestinal morphology is drastically altered following infection with ETEC and appears to be a less variable response criterion to evaluate. For each of these outcome variables, we have provided quantitative estimates of the responses seen across the literature as well as the variability within them. While there is a large degree of variability across ETEC challenge experiments, we have provided a quantitative summary of these studies and a Microsoft Excel-based tool was created to calculate sample sizes for future studies that can aid researchers in designing future work.

## Introduction

During the weaning transition, piglets undergo rapid changes environmentally, nutritionally, and immunologically (Zheng et al., 2021). These factors in combination with exposure to gastrointestinal pathogens can result in postweaning diarrhea (**PWD**), which is one of the most economically important issues for the swine industry due to increased mortality, suppressed growth rates, and costs associated with treatment (Bonetti et al., 2021). One of the most common etiological agents prompting PWD is enterotoxigenic *Escherichia coli* (**ETEC**), which are categorized according to virulence factors known as adhesins and enterotoxins (Smith and Linggood, 1971; Berberov et al., 2004; Zhang et al., 2007). Adhesins located on the bacterial fimbriae regulate the binding of the pathogens to epithelial cells and subsequent colonization within the intestine. The most common fimbriae associated with ETEC infection in postweaning pigs are F4 (K88) or F18. There are other fimbriae types such as F5 (K99), F6 (987P), F17, and F41, but they are commonly associated with neonatal diarrhea prior to weaning (Jin and Zhao, 2000). Upon fimbriae attachment and ETEC colonization, enterotoxins are produced which induce a rapid loss of electrolytes and secretion of fluid into the intestinal lumen, leading to the clinical observation of diarrhea (Nagy and Fekete, 2005). Heat labile (**LT**) and heat stable (STa, STb, and enteroaggregative heat-stable toxin 1 [**EAST1**]) are the two classes of enterotoxins produced by ETEC. Strains of ETEC with F4 (K88) fimbriae typically produce LT and STb (on some occasions STa may or may not be produced) enterotoxins upon bacterial adhesion and colonization, while strains exhibiting the F18 fimbriae will produce STa and STb toxins (Duan et al., 2012).

Also referred to as enteric colibacillosis, PWD caused by ETEC typically occurs within the first two weeks postweaning and results in watery, profuse diarrhea that can last anywhere from 1 to 5 d after infection (Sun and Kim, 2017). Subsequently, dehydration, reduced growth, and in some cases eventual mortality can occur (Verdonck et al., 2007). Prior to the establishment of the Veterinary Feed Directive in 2017, prevention or control of PWD caused by ETEC included therapeutic treatment with antibiotics, but today the addition of pharmacological levels of zinc oxide (ZnO) to the nursery diet is perhaps more common (Bonetti et al., 2021; Juhász et al., 2022). Due to concerns with antimicrobial resistance and excess zinc excretion in feces, there is strict consumer and regulatory pressure to limit this practice in both Europe and Canada (Commission, 2016). The demand for alternative strategies to combat PWD is growing in need and has become a focal point for many in the swine industry worldwide. Many proposed solutions include altering diet composition through lower crude protein (Bikker et al., 2006; Wellock et al., 2008), different fiber combinations (Htoo et al., 2007), and the inclusion of feed additives like diet acidifiers (Tsiloyiannis et al., 2001), pre- and probiotics (Hayakawa et al., 2016), nucleotides (Martinez-Puig et al., 2007), or essential oils (Tian and Piao, 2019). However, to effectively evaluate any intervention mechanism for PWD caused by ETEC, it is important to appropriately replicate ETEC infection by purposefully inoculating pigs with the pathogen to induce an experimental infection, known as an in vivo challenge model (Adewole et al., 2015).

Utilizing an ETEC challenge model in postweaning pigs has been demonstrated on numerous accounts; however, results from these studies are often extremely variable. A review by Luise et al. (2019b) summarized ETEC F4 and F18 infection models and focused on factors to consider when planning the challenge, such as genetic susceptibility to ETEC, preconditioning practices (i.e., administration of antibiotics or fasting prior to inoculation), and the timing and dose of ETEC inoculation. These authors also outlined methods to determine the effectiveness of an ETEC challenge based on diarrhea, rectal temperature, fecal bacterial shedding, immunoglobulin A, and intestinal mucosa ETEC receptor expression. Luise et al. (2019b) provided a great summary of features to consider when planning F4 or F18 challenge experiments in effort to decrease the variability in the challenge response. However, to our knowledge, there has been no attempt to provide quantitative assessment of this variability that can be useful to continue refining and standardizing ETEC infection studies. Since sample size calculations are often conducted using information extracted from published studies, the immense variability across the body of literature has perhaps resulted in subsequent sample sizes not truly reflective of what is required to generate a statistically significant outcome. Therefore, our objective was to review recent ETEC challenge experiments and provide quantitative measurements of the responses and variability among the most widely evaluated response criteria. Using this information, a second aim of this work was to generate a sample size calculation tool that accounts for this variability and can assist researchers with designing future ETEC challenge studies.

## Materials and Methods

The literature search was conducted utilizing PubMed (www.pubmed.ncbi.nlm.nih.gov), Web of Science (www.webofscience.com), and Scopus (www.scopus.com). The aim was to identify studies that challenged weanling pigs with ETEC F4 or F18. Search terms included a combination of the following: weanling pig OR nursery pig AND *Escherichia coli* OR ETEC F4 OR ETEC F18 OR ETEC K88 OR challenge. To narrow the search so that response criteria could be identified, a subset of search terms was included: growth OR immune OR fecal. Bibliographies from papers generated from the search were also scanned to identify relevant literature. Articles were restricted to peer-reviewed, in vivo studies, with English as the primary language, and published between the years of 2010 and 2022. This time frame was selected in effort to account for the vast changes in genetic composition, nutrition, health, and management seen in the swine industry over recent years. Additionally, these parameters allowed for enough data to be extracted without overlapping previously written reviews. Studies utilizing a lipopolysaccharide (**LPS**) challenge were not included. Once relevant papers were located, data were organized to identify the most frequently evaluated response criteria. Next, the means and measure of variability were extracted for each response criterion where quantitative values were provided (i.e., data presented as figures or charts with no numeric measure were not utilized) and summarized in a series of tables. For each response criteria, the mean and SD were presented by the type of ETEC utilized for the challenge, then chronologically according to the publishing date for each reference. Additionally, for each response the percent change between the control and treatment groups was calculated to provide an estimated magnitude of effect. The data from these tables were then organized in a literature database within a Microsoft Excel spreadsheet (Supplementary Data 1). This database was utilized to develop two calculators located within the Excel spreadsheet that use the mean and SD from the literature to determine the sample size needed to detect a significant difference between two treatment groups (calculator 1) or given a desired magnitude of difference (calculator 2). More detailed information regarding the sample size calculators is provided at the end of this review.

## Results and Discussion

### Considerations of the challenge model

The multidimensional nature of published ETEC infection models makes it difficult to directly compare studies. Several factors including the use of experimental controls, the age and genetic background of experimental animals, the timing and dosage of inoculation with ETEC, study length, response criteria measured and methodology to evaluate them, and the number of replicates are vastly different across the body of literature. The importance of experimental controls has long been understood (Boring, 1954); however, as it relates to ETEC challenge studies, the research question ultimately dictates the type of controls that will be used. If a researcher is interested in investigating how ETEC infection impacts the pig, then including a nonchallenged control group is critical. However, this adds another layer of complexity when designing the study, as strict biosecurity measures are needed to prevent the cross-contamination between treatments to keep the nonchallenged pigs healthy. Often times, this requires additional personnel or enhanced biosecurity level 2 (**BSL-2**) facilities with separate housing environments, which could limit the feasibility of the study. Conversely, if the researcher’s objective is to study the efficacy of an intervention strategy against ETEC infection, then perhaps a nonchallenged group of pigs may not be required. In this case, all pigs may be challenged with ETEC, but the use of a strong negative or positive control intervention, such as an antibiotic known to mitigate ETEC, would accomplish this objective. Among studies included in this review, 67% included a nonchallenged control group, and each of these papers provided statistical comparisons of nonchallenged pigs to pigs challenged with ETEC.

The dose and timing of inoculation with ETEC are both crucial parameters that are extremely variable across the studies presented in this review. For the sake of simplicity, we have displayed the inoculum dosages across this review as the total colony forming units (**CFUs**) administered to the animal, since many studies report the dosage of inoculum in mL and concentration on a per mL basis. The range of total CFUs administered to pigs to elicit a challenge is just one of many factors that makes comparison of challenge models difficult, and future work focusing on standardizing this dose should be done. Across the studies included in this paper, the range in dosage of ETEC F4 was from 1.5 × 10^5^ to 1.0 × 10^12^ CFUs, while F18 dosages varied between 2.0 × 10^9^ and 9.0 × 10^10^ CFU. It has been demonstrated that ETEC infection can occur with a single dose of approximately 10^9^ CFUs (Berberov et al., 2004; Wellock et al., 2008; Boeckman et al., 2022). However, it appears as though the type and dosage of ETEC play a role in the response observed, depending on the outcome variable being evaluated. This is discussed further in subsequent sections.

The timing of inoculation with ETEC is also important to consider and varies among studies. Based on our review of the literature and information presented in Luise et al. (2019b), inoculation appears to be most effective when it coincides with the expression of ETEC receptors in the piglets’ small intestine, which are impacted by age. According to Fairbrother et al. (2005), F4 fimbriae receptors within the piglet’s intestine are expressed from birth until maturity. Conversely, others have suggested that F18 receptors may not be fully expressed until closer to 18 to 20 d of age (Nagy et al., 1992; Nadeau et al., 2017). This indicates that administration of the inoculum during ETEC challenge studies should coincide with the time at which the small intestine has adequate receptivity for bacterial attachment. The weaning age of piglets within the included papers varied from 19 to 28 d of age, therefore we would expect to see expression of both ETEC F4 and F18 receptors by all pigs. The F4 and F18 strains of ETEC are commonly associated with PWD; therefore, it is important that ETEC challenge studies simulate the multiple stressors pigs undergo during the weaning transition which coincides with the incidence of PWD. Thus, across the tables in this review, the timing of inoculation is reported as the number of days postweaning (**dpw**), rather than the age of pigs at the time of inoculation. The amount of time that passes between the weaning transition and ETEC challenge may impact the response observed, making dpw a more suitable characteristic to compare studies.

Sample size is one of the most variable features of ETEC challenge studies. Determining the number of replicates needed per experimental group is a crucial component of designing a study (Wittes, 2002). Generally, sample sizes are calculated from previously published studies using the mean and measure of variability. Thus, the wide range of sample sizes utilized across ETEC studies is not surprising, given the extreme variation in responses that are reported across the literature. In the subsequent sections of this review, we summarize the types of responses and variability seen among several of the most widely evaluated response criteria in ETEC F4 and F18 challenge studies. Using data extracted from these ­papers, we have generated tables to display quantitative values for these responses that can assist researchers with sample size calculations for future studies.

### Growth performance

Maximizing growth performance in the postweaning period is of great importance yet is challenging due to low feed intake during the first 7 to 10 d after weaning. Proper development of the gastrointestinal (GI) tract during this time is critical, where the rate and efficiency of gain can be heavily impacted by GI health and function (Becker et al., 2020). Evaluating average daily gain (**ADG**), average daily feed intake (**ADFI**), and feed efficiency (**G:F**) are commonplace in swine nutrition research. However, when considering challenge studies, the nature of the research question ultimately dictates whether growth performance is a valuable response criterion to examine. For example, those investigating a nutritional intervention to ETEC infection may place greater emphasis on growth performance than researchers studying the effect of a vaccination program on immunological response. In the most practical sense, quantifying changes in growth performance following ETEC challenge can provide useful information to the swine industry, and collection of these data is inexpensive and rather simple compared to responses discussed through the remainder of this review. However, our findings indicate that there is a high degree of variability surrounding growth performance responses to both F4 and F18 ETEC.

Many factors can impact the magnitude of difference in growth performance between two treatment groups including the age of piglets at the time of inoculation and the dosage of inoculation. Furthermore, the statistical significance for that response is dependent upon the magnitude of response, variation, and number of replicates. Based on the studies reviewed, both a larger dose and a greater effect size were needed to observe a significant difference in ADG when pigs were inoculated with F18 ETEC compared to F4. A final inoculum concentration of 2 × 10^9^ CFU and a difference in ADG between treatment groups of approximately 21% was needed to detect statistical significance following an F18 challenge. Conversely, in F4 challenge studies, a dosage of 1 × 10^9^ CFU and a difference in ADG of 16% yielded statistical significance. It has been shown that expression of F4 receptors in the small intestine is earlier than that of F18 (Sun and Kim, 2017); however, it is difficult to confer whether this directly impacts the magnitude of response seen, given other factors of the challenge model can also play a role. Inoculum dose and the percent change between control and treatment groups did not play as large of a role in piglet responses in ADFI following ETEC challenge, and very few studies observed significant responses in G:F.

A goal of our review was to provide a summary of the variability around these response criteria to assist in sample size calculation for future studies. The importance of appropriate replication does not need reiterated but appears to be a factor that explains the variability in piglet growth performance responses following ETEC challenge. In a study by Koo et al. (2020), the authors challenged pigs with 5 × 10^10^ ETEC F4 and observed a 33% and 22% reduction in ADG and ADFI, respectively, but these differences were not statistically significant. Along with a relatively small sample size (*n* = 7 pigs per treatment), pigs in this study were challenged on d 10, but data collection postchallenge only lasted until d 13. Generally, in nursery nutrition studies, a longer data collection period would be preferred to ensure detection of statistically significant differences in performance, if present. However, since ETEC infection can manifest rather quickly, the negative ­impact of a short, transient infection would be more substantial compared to if the study was longer and there was opportunity for pigs to recover from the challenge. An ETEC F4 challenge study by Xu et al. (2020) used 1 × 10^10^ CFU and observed a 27% decrease in ADG with 6 replications per treatment and a 7-d data collection period, but again, these differences were not statistically significant. The number of replicates used across studies varies considerably. This is likely due to the immense variability in the type of growth performance responses reported as well as the variability within these responses across literature, which has made sample size calculation challenging. Table 1 summarizes the responses seen and variability surrounding ADG, ADFI, and G:F. The means and SD represent the period postinoculation from each study. Additionally, a percent change column was included for each response to understand the difference that was observed between the control and treatment groups for each study. It is important to note that not all papers included in this review utilized a nonchallenged control group. A greater proportion of studies reported a significant response in ADG and ADFI when compared to G:F. However, due to the relatively small sample size used in most ETEC challenge studies, interpretation of growth responses should be done carefully. Regardless, the importance of quantifying changes in growth performance due to ETEC challenge is apparent, especially when considering nutritional intervention strategies for PWD caused by ETEC. However, from our review of the literature, the concentration of inoculum and number of replications per treatment group appear to heavily impact the response seen.

**Table 1.** Summary of variability in growth performance responses following ETEC challenge in weanling pigs^1^

	Description of challenge model				ADG, g/d		ADFI, g/d		G:F	
Reference	ETEC type	Inoculum dose (CFUs)	Inoculation time (dpw)^2^	Study length (dpi)^3^	Mean ± SD	% Change^4^	Mean ± SD	% Change^4^	Mean ± SD	% Change^4^
Liu et al., 2010	F4	3 × 10^11^	7	7	265 ± 19.0^*^	−16	392 ± 76.8^*^	−7	0.67 ± 0.21^†^	−11
Nyachoti et al., 2012	F4	1 × 10^10^	8	7	161 ± 96.0	+79	230 ± 99.0	+50	0.72 ± 0.39	+20
Trevisi et al., 2011	F4	2 × 10^10^	7	5 to 7	138 ± 63.7^*^	−39	285 ± 36.1^*^	−16	—	—
Lee et al., 2012	F4	5 × 10^9^	14	14	449 ± 39.2^*^	−31	647 ± 203.3	−16	0.69 ± 0.09	−18
Wu et al., 2012	F4	5 × 10^9^	13	6	363 ± 18.1^*^	+18	595 ± 27.7^†^	+6	0.62 ± 0.03^*^	+13
Almeida et al., 2013	F4	9 × 10^10^	6	5	143 ± 180.2^*^	−42	591 ± 545.9	−12	0.27 ± 0.14	−32
Gao et al., 2013	F4	1 × 10^9^	1, 3, 5, 7	11	100 ± 48.9^*^	−29	280 ± 73.5	−13	0.37 ± 0.05^*^	−16
Khafipour et al., 2014	F4	6 × 10^10^	8 to 10	10	245 ± 69.8	−16	333 ± 36.7	−12	0.73 ± 0.22	−4
Yang et al., 2014	F4	1 × 10^9^	15	3	323 ± 147.0	−9	374 ± 36.7	−2	0.80 ± 0.47	−11
Li et al., 2015	F4	2 × 10^10^	14	27	400 ± 60.9	+8	598 ± 76.7^*^	+3	0.67 ± 0.05^*^	+5
Trevisi et al., 2015	F4	2 × 10^8^	7	14	201 ± 231.5	−5	—	—	—	—
Chen et al., 2016	F4	5 × 10^10^	7	7	198 ± 73.5	+8	351 ± 83.1	+24	0.56 ± 0.15^*^	+19
Han et al., 2016	F4	3 × 10^8^	0	7	142 ± 51.4^*^	−73	—	—	—	—
Lee et al., 2017	F4	3 × 10^10^	7	14	142 ± 59.4^*^	—	—	—	—	—
Lei et al., 2017	F4	5 × 10^10^	8 to 10	14	222 ± 8.9^*^	+44	298 ± 35.8^*^	+34	0.74 ± 0.07	+6
Pan et al., 2017	F4	1 × 10^11^	9	3	320 ± 45.8^*^	−21	391 ± 53.6^*^	−20	0.82 ± 0.05	−1
Koo et al., 2019	F4	5 × 10^10^	10	3	376 ± 150.7	−33	590 ± 182.2^†^	−22	0.67 ± 0.20^†^	+7
Lopez-Colom et al., 2019	F4	2 × 10^9^	7	8	117 ± 256.0	−9	211 ± 129.5	−4	—	—
Luise et al., 2019a, b	F4	2 × 10^5^	7	13	125 ± 94.2	+24	313 ± 148.0	+18	0.33 ± 0.35	−33
Choi et al., 2020	F4	5 × 10^7^	7	5	306 ± 149.4^†^	−46	559 ± 105.3	−15	—	—
Lei et al., 2020	F4	5 × 10^9^	22	2	428 ± 47.0^*^	−16	643 ± 40.3	−6	0.66 ± 0.08^*^	−10
Wang et al., 2020	F4	1 × 10^11^	15	5	334 ± 40.3	+20	830 ± 57.7^*^	+21	0.40 ± 0.29	−4
Xu et al., 2020	F4	1 × 10^10^	7	14	444 ± 83.8^*^	−27	729 ± 104.1^*^	−22	0.61 ± 0.05	−8
Yu et al., 2021	F4	1 × 10^12^	19	2	390 ± 36.6	−8	486 ± 19.6^*^	−8	—	—
Kim et al., 2019	F18	3 × 10^10^	7	11	214 ± 141.3^*^	−59	437 ± 201.9^*^	−36	0.46 ± 0.27	−33
Li et al., 2019	F18	2 × 10^10^	7	7	250 ± 94.8^*^	−41	682 ± 94.9^*^	−31	0.62 ± 0.18	−20
Wojnicki et al., 2019	F18	9 × 10^10^	13	10	505 ± 159.0	−10	1,282 ± 785.0	+3	0.39 ± 0.20^*^	−15
Becker et al., 2020	F18	1 × 10^10^	7	10	238 ± 31.6^*^	−51	335 ± 31.6^*^	−33	0.63 ± 0.19	−33
Duarte et al., 2020	F18	2 × 10^9^	13	13	355 ± 116^*^	−21	465 ± 82.0	−5	0.76 ± 0.16^*^	−17
Hong et al., 2021	F18	2 × 10^9^	7	13	335 ± 86.6^*^	+78	416 ± 96.7^*^	+70	0.82 ± 0.10	+1
Sun et al., 2021	F18	6 × 10^9^	7	12	435 ± 333.8	−3	664 ± 449.7	+0.39	0.65 ± 0.14	−5
Chang et al., 2022	F18	3 × 10^10^	7	14	251 ± 57.2^*^	−50	393 ± 31.7	−3	0.64 ± 0.12^*^	−49
F4 Average	—	9 × 10^10^	—	—	260 ± 86.3	—	436 ± 104.8	—	0.62 ± 0.17	—
F18 Average	—	3 × 10^10^	—	—	323 ± 127.5	—	584 ± 328.5	—	0.62 ± 0.17	—

^1^Mean ± SD represents the period reported immediately postinoculation from each study.

^2^Time of inoculation with ETEC, days postweaning (dpw).

^3^Length of time data was collected following inoculation with ETEC, days postinoculation (dpi).

^4^The calculated percent change between the control and treatment groups.

^*^Indicates that a statistically significant difference was observed between the control and treatment groups (*P* < 0.05).

^+^Indicates that a marginally significant difference was observed between the control and treatment groups (0.05 < *P* < 0.10).

### Fecal parameters

#### Fecal consistency

One of the most notable and easily detected clinical signs of ETEC infection is diarrhea, which can be defined as an increased frequency of defecation accompanied by feces which contain reduced dry matter content (Pedersen et al., 2011a). Diarrhea can last anywhere from 1 to 5 d following ETEC infection (Sun and Kim, 2017). In research settings, evaluation of fecal consistency as an indicator of diarrhea is a widely used tool to determine the effectiveness of the challenge model (Luise et al., 2019b). Generally, visual fecal scoring or analysis of fecal dry matter (**DM**) is utilized to assess fecal consistency; however, there is substantial variability in the methodologies used to evaluate these response criteria. Fecal scoring requires the subjective evaluation of the consistency of feces, usually per experimental unit, according to a Likert-type scale. Various fecal scoring scales have been implemented within the literature, but the majority of studies included in this review utilized one of two scoring systems: a 1 to 5 scale where 1 = normal feces and 5 = severe diarrhea, or a 0 to 3 scale where 0 = normal feces and 3 = severe diarrhea. Due to the subjective nature of visual fecal scoring, it is imperative that the same scorer(s) are utilized for trial entirety and that proper blinding of the fecal scorers to treatments is carried out as to avoid observation bias. Another challenge of visual fecal scoring is determining the threshold that dictates clinical diarrhea, which varies between studies depending on the fecal scoring scale that was used. For example, Sun et al. (2021) scored feces on a 0 to 3 scale, where fecal scores ≥1 were classified as diarrhea. Alternatively, Li et al. (2019) used a 1 to 4 scale for fecal consistency assessment and considered scores ≥3 as diarrhea. This difference in experimental procedures may seem small, but greatly contributes to the variability seen within literature as it relates to fecal consistency. In general, visual fecal scoring is subject to intra- and inter-observer variation (Pedersen and Toft, 2011), and the resulting data is inherently qualitative and ordinal in nature. When assessing a response criterion according to a scale like fecal scoring, the outcomes are not continuous or discrete, which can lead to inaccuracies in statistical analysis, where often these data are analyzed as if they were quantitative and normally distributed. White et al. (2016) reiterated the fact that analyzing qualitative variables, such as fecal scores, can be difficult and requires the appropriate use of inferential statistics to avoid reporting misleading results. Unfortunately, this mistake is commonly made in animal science research and was observed on nearly every account among the studies included in this review. Therefore, analysis of fecal DM can serve as a more objective measurement of fecal consistency and may be more straightforward to analyze statistically given it is quantitative and reasonably fits model assumptions for normality. This methodology involves the drying of fecal samples to calculate the amount of moisture extracted during the drying process, resulting in a final DM % of the sample (Pedersen et al., 2011b). Multiple protocols have been developed for evaluating fecal DM (Klopfenstein et al., 1995; Callan et al., 2007; Partanen et al., 2007) with varying degrees of repeatability and reproducibility. Of the studies reviewed, only one utilized the fecal DM approach, with all other studies implementing some degree of visual fecal scoring (Table 2). Application of fecal DM analysis may be limited due to necessary equipment to conduct analyses and time constraints for collection and drying of samples. Additionally, a pitfall of fecal DM analysis specific to weanling pig studies is the difficulty in collecting appropriate quantities of fecal material required for analysis, given the small amount of feces produced from a newly weaned pig.

**Table 2.** Summary of ETEC challenge studies evaluating fecal consistency as a response criterion

	Description of challenge model						Fecal Consistency Response^4^	
Reference	ETEC type	Inoculum dose (CFUs)	Inoculation time (dpw)^1^	Number of replicates	Method^2^	Collection time (dpi)^3^	Pre-inoculation	Postinoculation
Heo et al., 2010a, b	F4	1 × 10^8^	3 to 5	12	FS	0 to 14		↑
Liu et al., 2010	F4	3 × 10^11^	7	6	FS	0 to 7	—	↑
Trevisi et al., 2011	F4	2 × 10^10^	7	6	FS	1 to 7	—	ND
Lee et al., 2012	F4	5 × 10^9^	14	6	FS	0 to 14	—	↑
Nyachoti et al., 2012	F4	1 × 10^10^	8	9	FS	0 to 5	—	ND
Almeida et al., 2013	F4	9 × 10^10^	6	8	FS	0 to 5		ND
Gao et al., 2013	F4	1 × 10^9^	1, 3, 5, 7	6	FS	0 to 11		↑
Khafipour et al., 2014	F4	6 × 10^10^	8 to 10	5	FS	2, 4, 7	—	↑
Li et al., 2015	F4	2 × 10^10^	14	7	FS	7, 14, 21, 28	—	↓
Trevisi et al., 2015	F4	2 × 10^8^	7	10	FS	−12 hpi to 6	ND	↑
Chen et al., 2016	F4	5 × 10^10^	7	9	FS	1 to 5	—	↓
Han et al., 2016	F4	3 × 10^8^	0	6	FS	1 to 7	—	↓
Rhouma et al., 2016	F4	1 × 10^9^	7	12	FS	−3 to 35	ND	↑
Lee et al., 2017	F4	3 × 10^10^	7	8	FS	0 to 14	—	↑
Lei et al., 2017	F4	5 × 10^10^	7	5	FS	−7 to 21	↓	↓
Pan et al., 2017	F4	1 × 10^11^	9	6	FS	−9 to 12	ND	↑
Choi et al., 2020	F4	5 × 10^7^	7	6	FS	0 to 54 hpi^*^	—	↑
Wang et al., 2020	F4	1 × 10^11^	15	6	FS	0 to 6	—	↑
Lei et al., 2020	F4	5 × 10^9^	22	5	FS	−21 to 3	ND	↑
Kim et al., 2019	F18	3 × 10^10^	7	12	FS	0 to 11	—	↑
Li et al., 2019	F18	2 × 10^10^	7	10	FS	−7 to 7	ND	↑
Wojnicki et al., 2019	F18	9 × 10^10^	13	12 to 13	FS	−13 to 10	ND	↑
Becker et al., 2020	F18	1 × 10^10^	7	8 to 10	FS	0 to 10	—	↑
Duarte et al., 2020	F18	2 × 10^9^	7	8	FS	−3 to 20	ND	↑
Smith et al., 2020	F18	9 × 10^10^	10	18	DM	7	—	↑
Sun et al., 2021	F18	6 × 10^9^	7	8	FS	−11 to 25	ND	↑
Chang et al., 2022	F18	3 × 10^10^	4	9	FS	0 to 14	ND	ND
He et al., 2022	F18	6 × 10^10^	7	12	FS	0 to 21	—	↑

^1^Time of inoculation with ETEC, days postweaning (dpw).

^2^Method for evaluation of fecal consistency: FS = fecal scoring; DM = fecal dry matter (DM) analysis.

^3^Length of time data was collected following inoculation with ETEC, days postinoculation (dpi).

^4^Fecal consistency response following challenge with ETEC: ↑ indicates an increase in diarrhea; ↓ indicates a decrease in diarrhea; ND indicates no statistical difference in diarrhea.

^*^Hours postinoculation (hpi).

Again, the appropriate use of experimental controls is necessary to effectively evaluate fecal consistency as a response criterion. A nonchallenged control group is recommended for *in vivo* experiments (National Research Council, 2012), which would allow for direct comparison to groups challenged with ETEC. Table 2 displays ETEC challenge studies evaluating fecal consistency and their respective responses. Unfortunately, nearly all fecal scoring data is presented in the form of figures, and statistical information such as standard error of the mean are not provided, making it difficult to quantify the variability in fecal consistency responses seen within the literature. The ordinal nature of this data makes sample size calculation difficult, thus, fecal DM may be a more suitable response to evaluate. Despite only one ETEC challenge study using the fecal DM approach, there are other published papers in nonchallenged pigs where fecal DM analysis was conducted (Chance et al., 2021; Laskoski et al., 2021). Because these data are quantitative, they are typically presented in a form that allows scientists to extract measures of variability and means needed for sample size calculation. Despite being unable to summarize the variability in fecal consistency responses, it is important to note that only three papers included in this review failed to see a statistical difference in fecal consistency upon ETEC challenge. The body of literature strongly favors a worsening in fecal consistency when ETEC challenge is induced, and this response was reported as early as 6 h postinoculation (hpi; Lei and Kim, 2020), and lasted as long as 25 d postinoculation (dpi; Sun et al., 2021).

Additionally, it is important to consider the relationship between fecal consistency and other parameters, such as growth performance. It is well understood that PWD caused by ETEC can lead to subsequent reductions in growth performance (CITE). This was demonstrated across several studies included in this review, where an increase in diarrhea incidence postinoculation was accompanied by a statistically significant reduction in ADG (Liu et al., 2010; Lee et al., 2012; Gao et al., 2013; Pan et al., 2017; Kim et al., 2019; Li et al., 2019; Becker et al., 2020; Duarte et al., 2020; Lei et al., 2020; Chang et al., 2022). Utilizing fecal consistency as an indicator of ETEC challenge efficacy is valuable. However, there is a need for the standardization of the methodologies used for data collection and reporting of results such that statistical information is provided to assess variability for sample size calculation.

#### Fecal bacterial shedding

The normal microbiota–host relationship of the weanling pig prompts the shedding of multiple bacteria every day. In newly weaned piglets, infection with pathogenic bacteria such as ETEC causes an increase in the fecal shedding of that organism, which creates an environment for pathogen transmission to other animals via the fecal–oral route (Pluske, 2013). In the case of ETEC challenge studies, fecal bacterial shedding has been evaluated on several accounts, but with high degrees of variability in the methodologies used. Studies have investigated parameters such as total bacterial shedding (Wojnicki et al., 2019; Smith et al., 2020), total *E. coli* shedding (Li et al., 2015; Becker et al., 2020), F4- or F18-specific *E. coli* shedding (Hong et al., 2021), and hemolytic coliforms (He et al., 2022). Given *E. coli* shedding can occur even in healthy pigs, the most reliable evaluation of bacterial shedding as an indicator of ETEC infection is suggested to be specific to F4 or F18 strains. Typically, a 3 to 4 d measurement period postinoculation is required for proper adherence, colonization, and toxin production within the small intestine (Luise et al., 2019b). A notable difference among studies evaluating fecal bacterial shedding, regardless of the specific outcome variable they report, is the method by which bacteria are isolated from the feces. Wojnicki et al. (2019) and Smith et al. (2020) both utilized quantitative real-time polymerase chain reaction technology for fecal bacterial enumeration. Alternatively, studies by Becker et al. (2020), Li et al. (2015), and Li et al. (2019) quantified fecal bacteria via culture methods using selective media. Constraints related to experimental budgets, time, expertise, and required equipment may be the driving force behind which method of evaluation is used. While both procedures are widely accepted in literature, differences in the sensitivity and reproducibility of each procedure are important to consider quantified fecal bacteria via culture methods using selective media. Constraints related to experimental budgets, time, expertise, and required equipment may be the driving force behind which method of evaluation is used. While both procedures are widely accepted in literature, differences in the sensitivity and reproducibility of each procedure are important to consider (Lee et al., 2013). Regardless of the methodology used, the literature suggests that if ETEC infection is carried out effectively, the rate and number of bacteria shed in feces will increase. Perhaps the greatest difficulty in interpreting responses in fecal bacterial shedding stems from the presentation of data. The papers included in this review which evaluated fecal bacterial shedding provided data in the form of bacterial shedding scores, log_10_ CFU/g, and raw cycle threshold (**CT**) values, depending on the method of bacterial enumeration used. Directly comparing means and variability across studies is not justified and could lead to inaccurate interpretation of results. Additionally, like fecal consistency, these data are often presented in figure form to demonstrate the bacterial shedding over time, with no measure of variability provided. This causes the inability to utilize these published studies in power calculation, which is a severe pitfall of the body of literature. Table 3 provides a summary of the challenge models which quantified bacterial shedding and the response observed. Despite being unable to provide quantitative values for the variation in fecal bacterial shedding responses within these papers, Table 3 displays information that may be useful in the planning of future challenge work. Based on the information presented here and among other similar reviews, it appears that fecal bacterial enumeration should be conducted using PCR methods that allow for quantitative detection of F4- or F18-specific ETEC to accurately determine shedding of the pathogen.

**Table 3.** Summary of ETEC challenge studies evaluating fecal bacterial shedding as a response criterion

	Description of challenge model							Fecal bacterial shedding response^4^	
Reference	ETEC type	Inoculum dose (CFUs)	Inoculation time (dpw)^1^	Number of replicates	Method^2^	Outcome variable	Collection time (dpi)^3^	Pre-inoculation	Postinoculation
Trevisi et al., 2011	F4	2 × 10^10^	7	6	Culture	Total *E. coli*	10	—	ND
					PCR	F4 *E. coli*	10	—	ND
Lee et al., 2012	F4	5 × 10^9^	14	6	PCR	F4 *E. coli*	2, 7, 14	—	↑
Khafipour et al., 2014	F4	6 × 10^10^	8 to 10	5	Culture	Total *E. coli*	0, 11	ND	ND
					PCR	F4 *E. coli*	0, 11	ND	↑
Li et al., 2015	F4	2 × 10^10^	14	7	Culture	Total *E. coli*	0, 1, 28	ND	ND
Trevisi et al., 2015	F4	2 × 10^8^	7	10	Culture	F4 *E. coli*	0, 10	ND	↑
Chen et al., 2016	F4	5 × 10^10^	7	9	Culture	Total coliforms	7, 11	ND	ND
Han et al., 2016	F4	3 × 10^8^	0	6	PCR	F4 *E. coli*	1, 3, 7	—	↑
Rhouma et al., 2016	F4	1 × 10^9^	7	12	Culture	Total *E. coli*	−1, 1 to 36	ND	ND
					PCR	F4 *E. coli*		ND	↑
Lee et al., 2017	F4	3 × 10^8^	7	8	Culture	Total *E. coli*	1, 3, 7, 14	—	↑
Lei et al., 2020	F4	5 × 10^9^	22	5	Culture	Total coliforms	−1, 2	ND	↑
Kim et al., 2019	F18	3 × 10^10^	7	12	Culture	β-hemolytic coliforms	2, 5, 8, 11	—	↑
Li et al., 2019	F18	2 × 10^8^	7	10	Culture	Hemolytic *E. coli*	-7, 0, 1 to 3, 5, 7, 8	—	↑
Wojnicki et al., 2019	F18	9 × 10^10^	13	12 to 13	PCR	F18 *E. coli*	0, 5, 10	ND	↑
						Total *E. coli*	0, 5, 10	ND	↓
						Total bacteria	0, 5, 10	ND	ND
Becker et al., 2020	F18	1 × 10^10^	7	8 to 10	Culture	Total *E. coli*	0, 1 to 3, 5, 7, 10	ND	↑
Smith et al., 2020	F18	9 × 10^10^	10	18	PCR	Total bacteria	0, 7	↓	ND
						Total *E. coli*	0, 7	ND	↓
						F18 *E. coli*	0, 7	ND	↑
Hong et al., 2021	F18	2 × 10^9^	7	10 to 11	PCR	F18 *E. coli*	0, 7, 13	ND	ND

^1^Time of inoculation with ETEC, days postweaning (dpw).

^2^Method for evaluation of fecal bacterial shedding, polymerase chain reaction (PCR).

^3^Length of time data was collected following inoculation with ETEC, days postinoculation (dpi).

^4^Fecal bacterial shedding response following challenge with ETEC: ↑ indicates an increase in shedding; ↓ indicates a decrease in shedding; ND indicates no difference in shedding.

### Immunological

#### Immunoglobulins

Immunoglobulins play a pivotal role in the host defense against pathogens such as ETEC. Several immunoglobulins have been looked at as markers of ETEC infection in challenge studies; however, it is important to consider the nature of each immunoglobulin’s role in the humoral immune response during infection. Immunoglobulin A (**IgA**) is primarily found in bodily secretions such as saliva, milk, and as it relates to ETEC infection, intestinal fluid (Tizard, 2009). Secretory IgA (**sIgA**) is the most prevalent antibody of the intestine, protecting the intestinal tract from microbial colonization by preventing the adherence to the mucosal surface (Mantis and Forbes, 2010). Quantification of IgA has been done on multiple accounts (Table 4) with varying methodologies. Evaluation of sIgA must be done at the mucosal level, thereby requiring the euthanasia of the pig, so many studies evaluate circulating IgA in plasma or serum. Most studies included in this review analyzed IgA concentrations in the serum. The variability for serum IgA response was lower than IgA in the intestinal mucosa. Based on included papers, to elicit an IgA response with ETEC challenge, a final concentration of ETEC inoculum of at least 5 × 10^9^ CFU was required.

**Table 4.** Summary of variability in immunoglobulin responses following ETEC challenge in weanling pigs

				Response criteria^1^							
	Description of challenge model			IgA				IgG		IgM	
				Mucosal, µg/mg		Serum, mg/dL		Serum, mg/dL		Serum, mg/dL	
Reference	ETEC type	Inoculum dose (CFUs)	Number of replicates	Mean ± SD	% Change^2^	Mean ± SD	% Change^2^	Mean ± SD	% Change^2^	Mean ± SD	% Change^2^
Trevisi et al., 2011	F4	2 × 10^10^	6	—		53.8 ± 21.55^*^	32.1	—	—	—	—
Wu et al., 2012	F4	5 × 10^9^	8	0.08 ± 0.01	1.9	17.5 ± 1.58^*^	10.0	97.3 ± 8.38^*^	16.1	—	—
Li et al., 2015	F4	2 × 10^10^	7	—		43.9 ± 2.06^*^	9.8	212.0 ± 28.04^*^	19.7	22.4 ± 5.29^*^	53.7
Sugiharto et al., 2015	F4	5 × 10^7^	3 to 5	0.72 ± 0.39^*^	38.8	—	—	—	—	—	—
Trevisi et al., 2015	F4	1 × 10^8^	10	—	—	78.5 ± 32.25	11.1	—	—	—	—
Pan et al., 2017	F4	1 × 10^11^	6	0.13 ± 0.02^*^	96.7	—	—	—	—	—	—
Luise et al., 2019a,b	F4	2 × 10^5^	14 to 15	—	—	53.3 ± 34.90	52.6	1,470 ± 868.00	35.0	358.0 ± 209.0	21.2
Xu et al., 2020	F4	1 × 10^10^	6	—	—	109 ± 39.00	0.9	738.0 ± 120.02	10.2	78.0 ± 24.49	40.9
Becker et al., 2020	F18	1 × 10^10^	8 to 10	1.48 ± 0.96^*^	77.0	—	—	—	—	—	—
Hong et al., 2021	F18	2 × 10^9^	9	—	—	17.2 ± 7.42	3.0	486.4 ± 184.43^†^	15.9	85.9 ± 29.0	5.6
Chang et al., 2022	F18	3 × 10^10^	9	—	—	1.5 ± 0.33^*^	28.3	150.9 ± 26.43^*^	33.3	—	—
Median^3^	—	—	—	0.60 ± 0.35	—	46.8 ± 17.38	—	525.8 ± 175.15	—	136.08 ± 66.95	—

^1^Response criteria included immunoglobulin A (IgA), immunoglobulin G (IgG), and immunoglobulin M (IgM).

^2^The calculated percent change between the control and treatment groups.

^3^Median values were summarized at the bottom as the best measure of central tendency given the extreme outliers among data reported across studies.

^*^Indicates that a statistically significant difference was observed between the control and treatment groups (*P* < 0.05).

^†^Indicates that a marginally significant difference was observed between the control and treatment groups (0.05 < *P* < 0.10).

In addition to IgA, immunoglobulins G (**IgG**) and M (**IgM**) have also been evaluated as indicators of immune function in weanling pigs following ETEC challenge. Immunoglobulin G is the predominant immunoglobulin found in plasma, and the weaning transition can cause a 4-fold reduction in IgG levels within the pig (Butler et al., 2009). Next to IgG, IgM is the second most prevalent immunoglobulin in the serum and is produced in small amounts during a primary immune response (Tizard, 2009). While both immunoglobulins have been studied upon ETEC challenge within the literature, quantifying their levels postinoculation appears to be rather ineffective given they do not contribute directly to the host’s defense against bacterial adhesion at the mucosal level, when compared to IgA (Gaskins, 1998).

When evaluating immunoglobulins following ETEC challenge, consideration should be given to the time point at which sample collection will occur. Generally, once the animal has been exposed to a pathogen, it can take anywhere from 5 to 7 d for antibodies to appear, and typically, the peak of antibody production happens around 10 to 14 dpi (Tizard, 2009). Studies have reported responses in IgA production to ETEC as early as 7 dpi and lasting as long as 24 d (Nadeau et al., 2017; Laird et al., 2021). Among papers included in this review, the range in time points where a significant response in IgA was reported either in serum or the intestinal mucosa was rather large. Interestingly, Li et al. (2015) saw a significant increase in circulating IgA as soon as 24 hpi. Studies by Chang et al. (2022) and Trevisi et al. (2011) observed significant responses in IgA much later at 7 dpi. Both Becker et al. (2020) and Sugiharto et al. (2015) evaluated IgA in the intestinal mucosa and observed significant IgA changes at 10 and 8 dpi, respectively. It is apparent that evaluating immunoglobulins, specifically IgA, can serve as crucial indicators of ETEC infection. The timing of inoculation and the amount of time needed for peak activation of the host’s antibody defense system should be considered when evaluating these response criteria. While significant differences in IgA have been reported extremely early following an ETEC infection on rare accounts, it appears that analysis of IgA is most effective at least 7 dpi, and many authors continue evaluating IgA through later stages of infection, as late as 21 dpi. Additionally, the variability surrounding IgA within the literature can partially be attributed to differences in piglet age and laboratory procedures to quantify immunoglobulin presence in both serum and mucosa.

#### Pro-inflammatory cytokines

Pro-inflammatory cytokines are secreted proteins that act as signaling cells during an immune response, communicating with other immune cells of the body to target the pathogen upon infection (Zhang and An, 2007). Previous studies have focused heavily on the pro-inflammatory cytokines tumor necrosis factor-alpha (**TNF-α)** and interleukin-6 (**IL-6**), as they have been reported to be primary markers of pathogenic infection in the weanling pig (Zhang et al., 2010). Regulation and secretion of these cytokines is important for the pig to elicit an immune response upon ETEC infection; however, over-stimulation of these proteins, especially TNF-α, can result in reverse pathological effects that lead to inflammation. Therefore, the goal should be to decrease pro-inflammatory cytokine production following ETEC infection, thus, alleviating inflammation (Grimble, 1998). Ljuca et al. (2010) demonstrated that circulating cytokines in the serum are highly correlated to mucosal inflammation, indicating that these parameters could effectively be used to assess intestinal disease. Within the literature, TNF-α and IL-6 are the most predominantly studied pro-inflammatory cytokines as it relates to ETEC challenge trials, with most authors quantifying their presence in serum. Many studies reported a significant response in both parameters, but there is still a great deal of variability surrounding each response. In part, piglet age at the time of sample collection and methodologies to quantify the levels in serum most likely contribute to the variation that is seen within these outcome variables.

Generally, the enzyme-linked immunosorbent assay (**ELISA**) is used to quantify parameters such as pro-inflammatory cytokines, and this methodology was used across nearly all studies evaluating TNF-α and IL-6. While the ELISA is considered a reliable and effective measurement tool, considerations for differences in laboratory procedures could potentially explain variability in responses seen between studies. Similar to quantifying levels of immunoglobulins, it is nearly impossible to standardize all factors which can account for variability in the responses seen (i.e., age of piglets, laboratory techniques, or methods of evaluation) for immunological parameters. However, there is a need for research to focus on identifying optimal challenge model specifications, such as inoculum dose, timing of inoculation, duration of study, and genetic susceptibility of piglets, such that future experiments can follow a more standardized approach to ETEC infection. Based on the information we’ve summarized, assessment of TNF-α and IL-6 appear to be the most effective pro-inflammatory cytokines indicative of a response to ETEC. Evaluating these parameters can be done in both serum or mucosa, and most commonly using ELISA methods. Careful consideration of the large degree of variability should be done when conducting sample size calculations from previously published studies. The variability in both TNF-α and IL-6 responses among studies included in this review are presented in Tables 5 and 6, respectively.

**Table 5.** Summary of variability in tumor necrosis factor-alpha (TNF-α) responses following ETEC challenge in weanling pigs

				TNF-α			
	Description of challenge model			Mucosal, pg/mg		Serum, pg/mL	
Reference	ETEC type	Inoculum dose (CFUs)	No. of replicates	Mean ± SD	% Change^1^	Mean ± SD	% Change^1^
Xiao et al., 2014^2^	—	—	10	—	—	60.7 ± 5.40	9.0
Nyachoti et al., 2012	F4	1 × 10^10^	9	—	—	124.1 ± 28.20^*^	57.3
Lee et al., 2012	F4	5 × 10^9^	6	—	—	168.3 ± 6.73^*^	30.8
Li et al., 2015	F4	2 × 10^10^	7	—	—	154.0 ± 15.40^*^	15.3
Lee et al., 2017	F4	3 × 10^10^	8	—	—	93.2 ± 20.36^*^	64.2
Lei et al., 2020	F4	5 × 10^9^	5	—	—	84.8 ± 32.42^*^	66.7
Wang et al., 2020	F4	1 × 10^11^	6	—	—	182.4 ± 23.83^*^	46.5
Xu et al., 2020	F4	1 × 10^10^	6	—	—	86.7 ± 15.41^*^	19.6
Duarte et al., 2020	F18	2 × 10^9^	8	1.00 ± 0.31^†^	15.2	—	—
Smith et al., 2020	F18	9 × 10^10^	18	—	—	235.0 ± 184.70^*^	25.3
Hong et al., 2021	F18	2 × 10^9^	9	—	—	88.9 ± 31.73	1.1
Sun et al., 2021	F18	6 × 10^9^	8	0.88 ± 0.65^*^	29.7	60.5 ± 18.18^*^	25.1
Chang et al., 2022	F18	3 × 10^10^	9	—	—	33.1 ± 6.30^*^	32.5
Average	—	—	—	0.94 ± 0.48	—	114.3 ± 32.38	—

^1^The calculated percent change between the control and treatment group.

^2^ Xiao et al. (2014) did not report the specific strain of ETEC or the dose of ETEC inoculum utilized.

^*^Indicates that a statistically significant difference was observed between the control and treatment groups (*P* < 0.05).

^†^Indicates that a marginally significant difference was observed between the control and treatment groups (0.05 < *P* < 0.10).

**Table 6.** Summary of variability in interleukin-6 (IL-6) responses following ETEC challenge in weanling pigs

				IL-6			
	Description of challenge model			Mucosal, pg/mg		Serum, pg/mL	
Reference	ETEC type	Inoculum dose (CFUs)	No. of replicates	Mean ± SD	% Change	Mean ± SD	% Change
Xiao et al., 2014^1^	—	—	10	—	—	65.3 ± 5.72	10.2
Nyachoti et al., 2012	F4	1 × 10^10^	9	—	—	63.5 ± 21.00^*^	53.4
Lee et al., 2012	F4	5 × 10^9^	6	—	—	17.0 ± 3.16^*^	16.0
Wu et al., 2012	F4	5 × 10^9^	8	—	—	3.9 ± 0.25^*^	36.2
Li et al., 2015	F4	2 × 10^10^	7	—	—	25.7 ± 4.05^†^	27.0
Che et al., 2017	F4	1 × 10^9^	9	—	—	58.5 ± 4.32^*^	38.6
Lei et al., 2020	F4	5 × 10^9^	5	—	—	65.5 ± 30.19^*^	62.9
Wang et al., 2020	F4	1 × 10^11^	6	—	—	12.8 ± 0.73^*^	14.2
Xu et al., 2020	F4	1 × 10^10^	6	—	—	116.4 ± 7.27	8.2
Chang et al., 2022	F18	3 × 10^10^	9	—	—	46.7 ± 13.08^*^	41.9
Becker et al., 2020	F18	1 × 10^10^	8 to 10	0.6 ± 0.63	47.8	—	—
Duarte et al., 2020	F18	2 × 10^9^	8	3.6 ± 1.30^*^	44.7	—	—
Average	—	—	—	2.1 ± 0.97	—	47.53 ± 8.98	—

^1^ Xiao et al. (2014) did not report the specific strain of ETEC or the dose of ETEC inoculum utilized.

^*^Indicates a statistically significant response was observed (*P* < 0.05).

^†^Indicates a marginally significant response was observed (0.05 < *P* < 0.10).

### Intestinal morphology and permeability

It is well understood that during the weaning transition, villi atrophy can occur, resulting in less mucosal surface area available for absorption of nutrients or for proteins like sIgA to bind to (Pluske, 2013). Evaluation of small intestinal morphology can be a beneficial measurement to assess following ETEC challenge, especially when nutritional intervention is expected to alleviate the detrimental effects that bacterial adhesion and toxin release can have on the intestinal mucosa. However, euthanasia and necropsy are required to obtain such measurements; therefore, limiting the ability of some researchers to assess these outcome variables. There are numerous reports in the literature of changes in small intestinal morphology postinoculation with ETEC. Table 7 summarizes the studies included in this review that evaluated changes in small intestinal morphology. Assessment of morphology includes quantifying the villus height (**VH**), crypt depth (**CD**), and the VH:CD ratio by staining intestinal tissue with hematoxylin and eosin, where a larger VH and smaller CD are indicative of a more favorable morphological environment. It has been reported that ETEC infection can have detrimental effects on the small intestinal morphology, subsequently increasing intestinal permeability (Rong et al., 2015). This type of response was common in the reviewed papers, where ETEC infection, regardless of inoculum dose, timing, or other factors impaired villi height (Becker et al., 2020; Choi et al., 2020; Duarte et al., 2020). Interestingly, very few authors reported a significant response in CD postinoculation. This is supported by Al Masri et al. (2015), who reviewed piglet small intestinal morphology surrounding weaning and suggested there was no clear evidence that CD changes immediately postweaning or upon pathogenic infection. Including small intestinal morphology as outcome variables appear to be valuable tools to help assess the impacts of ETEC on the weanling pig, especially in studies investigating nutritional interventions that show promise to alter the intestinal morphology postweaning. Given morphological changes can occur around weaning even in clinically healthy pigs, the use of nonchallenged control pigs would be beneficial when assessing small intestinal morphology following ETEC challenge to determine if changes in morphology are attributed to ETEC (Table 8).

**Table 7.** Summary of variability in small intestinal morphology responses after ETEC infection^1^

Location	Ileum			Duodenum			Jejunum		
Measurement	Villus height, µm	Crypt depth, µm	VH:CD	Villus height,µm	Crypt depth, µm	VH:CD	Villus height, µm	Crypt depth, µm	VH:CD
Reference									
Trevisi et al., 2011	217 ± 34^*^	198 ± 23^*^	1.12 ± 0.19^*^	276 ± 34^*^	240 ± 23^*^	1.19 ± 0.19^*^	255 ± 34^*^	192 ± 23^*^	1.34 ± 0.19^*^
Nyachoti et al., 2012	403 ± 30^*^	278 ± 54	1.45 ± 0.30^*^	—	—	—	—	—	—
Wu et al., 2012	390 ± 35^*^	215 ± 16	1.84 ± 0.11^*^	435 ± 26	223 ± 13	1.93 ± 0.59	434 ± 45	226 ± 17^*^	1.94 ± 0.02^*^
Almeida et al., 2013	300 ± 26	223 ± 24^*^	1.36 ± 0.23	374 ± 60	266 ± 110	1.54 ± 0.74	—	—	—
Yang et al., 2014	565 ± 108	300 ± 54	1.90 ± 0.39	438 ± 78^*^	359 ± 56^*^	1.29 ± 0.34^*^	475 ± 88^*^	275 ± 27^*^	1.86 ± 0.54^*^
Pan et al., 2017	341 ± 33^*^	223 ± 22	1.54 ± 0.15^*^	426 ± 50^*^	244 ± 32	1.76 ± 0.27^*^	408 ± 41^*^	229 ± 26	1.79 ± 0.22^*^
Kim et al., 2019	365 ± 43	215 ± 22	1.70 ± 0.13	441 ± 68	307 ± 66	1.44 ± 0.14	395 ± 116^*^	241 ± 82	1. 64 ± 0.30
Becker et al., 2020	265 ± 39^*^	178 ± 31^†^	1.51 ± 0.29^*^	—	—	—	—	—	—
Choi et al., 2020	—	—	—	—	—	—	449 ± 95^*^	262 ± 42	1.98 ± 0.56
Duarte et al., 2020	—	—	—	—	—	—	395 ± 72^*^	275 ± 30^*^	1.48 ± 0.25^*^
Koo et al., 2020	394 ± 53	208 ± 27	1.91 ± 0.25	456 ± 62^*^	247 ± 34	1.87 ± 0.28^*^	444 ± 57	208 ± 25^*^	2.69 ± 0.25^*^
Lei et al., 2020	379 ± 64^*^	226 ± 35	1.70 ± 0.34^*^	475 ± 71^*^	224 ± 42	1.43 ± 0.45^*^	344 ± 69	225 ± 28	1.51 ± 0.47
Sun et al., 2021	314 ± 82	258 ± 52^*^	1.22 ± 0.17	—	—	—	314 ± 56^*^	231 ± 24^*^	1.80 ± 0.20^*^
Chang et al., 2022	351 ± 31^*^	159 ± 27^†^	2.28 ± 0.44^*^	—	—	—	—	—	—
Average	364 ± 52	224 ± 34	1.62 ± 0.26	420 ± 57	267 ± 39	1.53 ± 0.33	389 ± 70	237 ± 33	1.80 ± 0.31

^1^Means and SD represent the period reported immediately postinoculation for each study.

^*^Indicates that a statistically significant difference was observed between the control and treatment groups (*P* < 0.05).

**Table 8.** Summary of variability in tight junction gene expression in the ileum and jejunum of weanling pigs following ETEC challenge^1^

				Response criteria^2^					
Location				Ileum			Jejunum		
	Description of challenge model			OCLN	CLDN1	ZO-1	OCLN	CLDN1	ZO-1
Reference	No. of replicates	ETEC type	Inoculum dose (CFUs)						
Yang et al., 2014	6	F4	1.00 × 10^10^	0.91 ± 0.42	1.24 ± 0.56	1.56 ± 0.59	0.84 ± 0.29^*^	0.83 ± 0.73	0.64 ± 0.20^*^
Li et al., 2019	10	F18	2.10 × 10^10^	1.48 ± 1.68	1.44 ± 2.94^*^	1.37 ± 0.85	—	—	—
Becker et al., 2020	8 – 10	F18	1.14 × 10^10^	0.57 ± 1.07^*^	0.82 ± 0.59	0.76 ± 0.28^*^	—	—	—
Choi et al., 2020	6	F4	5.00 × 10^7^	—	—	—	0.85 ± 0.37	0.51 ± 0.20^†^	0.79 ± 0.34
Kim et al., 2019	12	F18	3.00 × 10^10^	—	—	—	1.32 ± 0.81	1.27 ± 1.52	1.42 ± 1.10^*^
Average	—	—	1.45 × 10^10^	0.99 ± 1.06	1.17 ± 1.36	1.23 ± 0.57	1.01 ± 0.49	0.87 ± 0.82	0.95 ± 0.55

^1^Data are presented as the mean ± SD in fold change for each response criteria.

^2^Response criteria included occludin (OCLN), claudin-1 (CLDN1), and zonula occludens-1 (ZO-1).

^*^Indicates a statistically significant difference was observed between the control and treatment groups (*P* < 0.05).

^†^Indicates a marginally significant difference was observed between the control and treatment groups (0.05 < *P* < 0.05).

Aside from the morphological changes, ETEC infection is also suspected to alter the permeability of the small intestine (Kim et al., 2022). Within the lumen of the small intestine resides a layer of epithelial cells that serve as the first line of defense against pathogens. Together, these epithelial cells form a barrier that is maintained by the connecting enterocytes, where specialized junctions known as tight junctions (**TJs**) seal the intercellular spaces between them (Mukiza and Dubreuil, 2013). These TJs are made up of a protein complex consisting of occludin (**OCLN**), zonula occludens protein-1 (**ZO-1**), and members of the claudin (**CLDN**) family of proteins (Puthenedam et al., 2007). Upon ETEC infection, TJs work to maintain epithelial barrier integrity in effort to prevent the passage of endotoxins across the intestinal brush border. This is generally accompanied by alterations in the expression of genes related to TJ proteins. Very few studies evaluated these outcome variables following ETEC infection and reported variable results. Becker et al. (2020) quantified the mRNA abundance of OCLN, ZO-1, and claudin-1 (**CLDN-1**) in the ileum of pigs at 10 dpi and reported elevated expression of OCLN and ZO-1 in unchallenged pigs compared to those challenged with ETEC, but no response in CLDN1 expression. Conversely, Li et al. (2019) evaluated these parameters 7 dpi and observed a significant increase in CLDN1 and no evidence of differences in OCLN or ZO-1. Work by Choi et al. (2020) found no change in OCLN and ZO-1 postinoculation and only a tendency for elevated CLDN1, while Kim et al. (2019) saw increased ZO-1 and no difference in OCLN or CLDN1 in the jejunum of pigs. Other techniques to measure epithelial transport more directly include ex vivo experiments such as Ussing chambers or in vivo assessments via orally administered markers of gut permeability (McLamb et al., 2013; Yang et al., 2014). While it is well understood that ETEC infection can have detrimental impacts on small intestinal morphology and permeability (Kim et al., 2012; Jayaraman et al., 2017; Sun et al., 2017), emphasis on alterations in TJ proteins is needed moving forward, considering the relatively low number of studies evaluating these response criteria and the high degree of variability among published literature.

### Development of a sample size calculation tool

After identifying the pertinent response criteria to include in ETEC challenge studies and providing quantitative measures of their responses and variability, our goal was to create a tool that could help researchers effectively compute sample size requirements for future studies using the information compiled in this review. The values for the means and SD from each study and every response criterion described throughout this paper were filed into a Microsoft Excel database. From there, two calculators were built to determine the number of replications needed to detect a statistically significant difference between two treatment groups or based on a specified magnitude of difference. These calculators can be found in Supplemental Data 1, where the first tab of the tool provides instructions for its use. However, to better outline how this tool can be used, two example scenarios are featured below.

#### Scenario 1 (sample size required to detect a difference between two groups)

Calculator 1 allows you to determine the number of replicates needed per treatment group to detect a significant difference between two groups. This calculator uses the mean and SD values from the ‘Literature Database’ tab, which coincide with the values presented in tables throughout this review. To begin, you should thoroughly read the information found on the “Instructions” tab, and then click on the tab labeled “Calculator 1” to begin your analysis. For this example, we will assume the following:

- The ETEC strain being used for the challenge is F18 (cell D8)
- The significance level (α) will be set at 0.05 (cell D9)
- The power (1 -β) will be set at 80% (cell D10)
- The control group means will be pulled from column H (Mean Value from Literature)
- We want to determine the number of replicates required to detect a significant difference in ADG
- The difference indicative of a biologically relevant effect in ADG has been determined to be 35% following challenge with ETEC F18

The steps to utilize this calculator to generate the required sample size given this scenario are as follows:

1. Select “F18” as the ETEC type (cell D8)
2. Select “0.05” as the α value (cell D9)
3. Select “80%” as the Power value (cell D10)
4. Copy the “Mean Value from Literature” for ADG of 303 (cell H17) to your “Control Mean” (cell D17)
5. Using the magnitude of effect of 35% as declared above, the value for the “Treatment Group” (cell E17) would be 408

The tool will then calculate the number of replicates required to detect a statistically significant difference between these two groups in the “Replicates Per Treatment Needed” area (cell F17). The calculation for this tool is derived from Dohoo et al. (2003) and is described below


$$\begin{array}{l} n\;=2\;\times \;\left[\frac{{\left(Z_{\frac{\alpha }{2}}-\;Z_{\beta }\right)}^{2}\times \;\sigma^{2}}{{\left(\mu_{1}-\;\mu_{2}\right)}^{2}}\right] \end{array}$$

Where *n* represents the sample size, $Z_{\alpha /2}$ represents the *Z* critical value for the significance level (α; the probability of a type I error), $Z_{\beta }$represents the *Z* critical value for beta (β; the probability of a type II error), $\sigma^{2}$represents the SD, $\mu_{1}$represents the control group mean, and $\mu_{2}$represents the treatment group mean. In this example, the number of replicates required per treatment group to detect a statistically significant difference in ADG would be 29.

#### Scenario 2 (sample size required to detect a significant difference given an effect size)

Calculator 2 allows you to determine the number of replicates required per treatment group given a specified magnitude of effect (i.e., the difference between the control and treatment groups). Since many ETEC challenge studies are conducted in specialized BSL-2 facilities, oftentimes there can be constraints on the experimental design due to space availability. In this case, it is critical to understand what the minimum number of replications would be to detect a statistically significant difference in the response criteria you are evaluating. Calculator 2 utilizes a desired magnitude of difference to provide sample size estimates for a series of effect sizes, which helps the user visualize the minimum difference that would be required to see a response. Additionally, this calculator allows the user to determine the maximum number of possible treatment groups that could be utilized in a study given a specified number of pens (assuming that pen is the experimental unit; regardless of if pigs are housed individually or as a group). Again, this calculator uses the mean and SD values from the “Literature Database” tab, which coincide with the values presented in tables throughout this review. To begin, you should thoroughly read the information found on the “Instructions” tab, and then click on the tab labeled “Calculator 2” to begin your analysis. For this example, we will assume the following:

- The ETEC strain being used for the challenge is F4 (cell D6)
- The significance level (α) will be set at 0.05 (cell D7)
- The power (1-β) will be set at 80% (cell D8)
- The control mean and SD will be pulled from the “Literature Database” tab
- We want to determine the number of replicates required to detect a significant difference in ADFI
- The difference indicative of a biologically relevant effect in ADFI has been determined to be 15% following challenge with ETEC F4
- In our experimental facility, we have a total of 40 pens to individually house animals (i.e., the animal/pen will be the experimental unit)

The steps to utilize this calculator to generate the required sample size given this scenario are as follows:

1. Select “F4” as the ETEC type (cell D6)
2. Select “0.05” as the α value (cell D7)
3. Select “80%” as the Power value (cell D8)
4. Select the response criterion of choice (cell D9)
5. The control mean (cell D10) and SD (cell D11) will auto-populate based on the information selected above
6. Enter the desired magnitude of difference of 15% (cell D12)
7. Enter the number of pens as 40 (cell D16)

The tool will provide sample size calculations for 10 different effect sizes. The initial magnitude of difference was set at 15% in cell D12, however, in cells D21 to M21 the range of effect sizes will be calculated. From there, the tool will hold the control mean constant (cells D19 to M19) and calculate the required treatment mean based on the magnitude of difference needed (cells D20 to M20). Next, the number of replicates required per treatment will be calculated in cells D23 to M23 and are highlighted in green. Additionally, the total number of replicates needed will be calculated in cells D24 to M24, assuming two treatment groups. Finally, based on the number of pens that were provided in cell D16, the tool will calculate the maximum number of possible treatments that can be used given these experimental constraints. These values will ­calculate in cells D25 to M25 and any nonfeasible values will highlight in red, meaning that the sample size required to detect a statistically significant difference is not feasible given the facility constraints you provided.

This calculator uses the same equation by Dohoo et al. (2003) as mentioned above. For this example, the number of replicates required per treatment group to detect a statistically significant difference in ADFI in a facility with 40 pens and an expected magnitude of difference between the control and treatment groups of 15% would be 130 replicates per group. As the magnitude of differences increases from 15% to 150% (cells D21 to M21), the number of replicates required per treatment group becomes smaller. As indicated by the red “n/a” in cell D25, based on the assumptions provided above, we would not have enough statistical power in this facility to see a statistically significant response in ADFI.

## Conclusions

In summary, there is considerable variation in piglet responses to ETEC F4 and F18 infection during challenge experiments. While this review summarized the most frequently evaluated response criteria used during these types of studies and quantified the variability around them; there is still a large magnitude of work needed to standardize a repeatable and reproducible challenge model for ETEC. Many factors contribute to the high degree of variation observed in the literature, which includes, but is not limited to the number of experimental animals needed, the precise dosage and timing of ETEC inoculation, and animal selection criteria. Additionally, there are many other outcome variables that have been studied, but were not included in this review, as their application was more limited. Here we have used the information from this review to quantitatively summarize the responses and variability from ETEC challenge models and generate a tool to allow for more accurate and simplified sample size calculations for future work. While this tool can provide value, it is important to consider it does not include all published ETEC data, nor does it account for external factors such as laboratory practices that could perhaps impact variability of response seen across studies. The complexity of ETEC studies and the inherent difficulty in utilizing their data to guide future models is ever-changing and requires constant reassessment. Additionally, further research surrounding experimental infection with ETEC in the weanling pig, specifically aiming to standardize infection models and reduce between-experiment variability, is needed to more effectively investigate nutritional or management strategies to mitigate PWD caused by ETEC.

## Supplementary Material

txad083_suppl_Supplementary_Data
